# A legume biofortification quandary: variability and genetic control of seed coat micronutrient accumulation in common beans

**DOI:** 10.3389/fpls.2013.00275

**Published:** 2013-07-29

**Authors:** Matthew W. Blair, Paulo Izquierdo, Carolina Astudillo, Michael A. Grusak

**Affiliations:** ^1^Department of Plant Breeding and Genetics, Cornell UniversityIthaca, NY, USA; ^2^CENICAÑA – Centro Nacional de Investigación en CañaCandelaria, Valle de Cauca, Colombia; ^3^Department of Agronomy, Michigan State UniversityEast Lansing, MI, USA; ^4^Department of Pediatrics, USDA-ARS Children's Nutrition Research Center, Baylor College of MedicineHouston, TX, USA

**Keywords:** advanced backcross breeding method, cotyledon, embryo axis, iron and zinc concentration, use of wild beans, seed coat

## Abstract

Common beans (*Phaseolus vulgaris* L.), like many legumes, are rich in iron, zinc, and certain other microelements that are generally found to be in low concentrations in cereals, other seed crops, and root or tubers and therefore are good candidates for biofortification. But a quandary exists in common bean biofortification: namely that the distribution of iron has been found to be variable between the principal parts of seed; namely the cotyledonary tissue, embryo axis and seed coat. The seed coat represents ten or more percent of the seed weight and must be considered specifically as it accumulates much of the anti-nutrients such as tannins that effect mineral bioavailability. Meanwhile the cotyledons accumulate starch and phosphorus in the form of phytates. The goal of this study was to evaluate a population of progeny derived from an advanced backcross of a wild bean and a cultivated Andean bean for seed coat versus cotyledonary minerals to identify variability and predict inheritance of the minerals. We used wild common beans because of their higher seed mineral concentration compared to cultivars and greater proportion of seed coat to total seed weight. Results showed the most important gene for seed coat iron was on linkage group B04 but also identified other QTL for seed coat and cotyledonary iron and zinc on other linkage groups, including B11 which has been important in studies of whole seed. The importance of these results in terms of physiology, candidate genes and plant breeding are discussed.

## Introduction

Biofortification is a relatively recent addition to breeding goals in plants based on improving the nutritional quality of the edible portion of the plant through traditional or transgenic approaches (Dwivedi et al., 2012). To date, most biofortification work has concentrated on micronutrients and vitamins, although conceivably protein content, amino acid distribution and beneficial secondary metabolites could be considered to be goals of biofortification (Welch, 1999). The stated goals of most biofortification work is to reduce mineral or vitamin deficiencies and where needed protein deficiencies. These deficiencies manifest themselves in conditions of iron deficiency anemia (IDA) but are not in themselves actual diseases but rather imbalances in the diet that need to be approached through modifications in the diet (Pfeiffer and McClafferty, 2007).

Micronutrient deficiencies are predicted to affect half the world's human population, with IDA being an especially common health concern affecting at least 2 billion people. IDA is caused by low consumption of iron especially in reproductive age women and developing adolescents (Welch, 1999). Zinc deficiency is suspected to be equally as common but has not been as well documented as IDA (Welch and Graham, 2002). While IDA causes losses in work productivity and developmental problems, zinc deficiency causes lowered disease immunity and stunting. These types of deficiencies are sometimes difficult to address through supplementation or fortification for technical, societal or economic reasons and therefore these minerals do not always reach the poor consumer (Dwivedi et al., 2012). How to reach the bottom of the pyramid of societies' economic strata (people making less than 2 dollars a day) with food containing sufficient micronutrients has been debated but one way is through biofortification of staple crops that are consumed in high amounts by the poor, such as rice, wheat and beans (seed crops) or potato and cassava (root and tuber crops).

Common bean is a highly nutritious food because of its balance of carbohydrates to protein (between 4:1 and 3:1), content of important vitamins and its micronutrient concentration, especially for iron which is at much higher levels than in the starchy staples of barley, corn or wheat. Common bean is widely grown in many parts of Asia, Africa, Europe and North, Central and South America and has two major types, the large seed Andean beans and the small seed Mesoamerican beans (Broughton et al., 2003). Consumption of common beans generally is higher than 20 to 30 kg/year in rural northeast Brazil, Central America and Mexico and can be as high as 40 to 60 kg/year in regions where meat is scarce such as in the Great Lakes of Africa. In Europe and North America consumption is low at 5 kg/year or less. Like other legumes, common bean is usually combined in the diet with a starch based food either in a mixture of two components (pulse + grain) or as a side dish such as “dals” made of lentils or “Frijoladas” made of beans. In some places common bean is the national dish as in Brazil where “Feijoada” is served daily or in the Dominican Republic where the “Bandera” is a twice-a-day meal made of rice with a side of beans. Certain regions of Central and South America consume mashed or re-heated “Calentao” beans as breakfast.

One reason for the micronutrient density of legumes is their anatomical seed structure, where a thick, maternally-derived seed coat or testa surrounds the expanded cotyledons of a quiescent but fairly large and well developed embryo. This structure differs radically from cereals where a thin, maternally-derived aleurone layer surrounds specialized endosperm tissue with a less well developed embryo in the bran. Breeding programs for cereals have had little concern for the sub-compartmentalization of micronutrients given that the bran is removed in milling processes and the major target tissue is the endosperm. However, in legumes, for the most part, the seed is consumed whole after a process of boiling, rather than by any processes of milling or grinding. In addition, the seed coat makes up 7 to 10% or more of the total seed weight of beans and is a source of consumer preference, the cotyledon makes up 85% or more of total seed weight and the embryo is only 2 or 3% of seed weight but is dense in nutrients (Ariza-Nieto et al., 2007).

Seed size varies more than seed coat thickness so smaller seeded legumes especially wild relatives of beans tend to have even higher percentages of seed coat compared to cotyledonary tissue of cultivated beans. The presence of these different seed tissues means that all three components of the common bean seed should be targets of biofortification: seed coat, cotyledons and embryo. An understanding of mineral distribution, loading and inheritance into each tissue is essential for making progress in breeding of this crop.

The goal of this research, therefore, was to evaluate the concentration of iron and zinc, in particular, and other minerals more generally in seed coats that were separated from cotyledons for a common bean population derived from a wild (small-seeded) common bean crossed with a cultivated (large-seeded) common bean. This analysis was part of a biofortification breeding program using the advanced backcross (AB) quantitative trait loci (QTL) approach as outlined in Tanksley and Nelson (1996); Blair et al. (2006) and Blair and Izquierdo (2012). The identification of QTL for seed coat *versus* cotyledonary mineral concentrations can be used in molecular breeding of common bean as regular peeling of seed coats for analysis is time consuming and onerous for the researcher. In addition, we were interested in determining the value of wild beans as a source of higher iron concentration as they have been found to have very high levels of this mineral (Guzmán-Maldonado et al., 2004) but have a disadvantage of small seed size.

## Materials and methods

### Seed source and field conditions

An advanced backcross (AB) population of 138 BC_2_F_2:5_ lines (cross NH 21154_ (F_2_)−1−M−M) was developed by crossing the cultivated recurrent parent Cerinza with the wild donor parent G10022, a source of high seed mineral concentration as described in Blair and Izquierdo (2012). Cerinza as the recurrent or recipient parent is large-seeded and of red color, and is rounder than a kidney bean.

Cerinza, like other Andean large-seeded beans, weighing approximately 50 g *per* 100 seed, has a good baseline of iron concentration averaging 60 ppm in multiple field trials (Astudillo and Blair, 2008). Meanwhile, the wild donor parent G10022 from Mexico is small seeded, weighing approximately 5 g *per* 100 seed and was discovered in a screening program for nutritional traits in wild beans and was found to have a very high concentration of 110 ppm iron concentration (Islam et al., 2002). Zinc concentration was less contrasting in previous studies of the two parents with G10022 having 38 ppm and Cerinza having 25 to 27 ppm.

The advantage of using the AB population instead of a simple cross between wild and cultivated beans for the nutritional analysis was that most of the genotypes were of similar seed size and adaptation (Blair et al., 2006; Blair and Izquierdo, 2012). They could therefore be grown together in the same field experiment at a site in Darién, Colombia (3°54′N, 76°30′W, 1485 m above sea level, average yearly temperature 20°C, average relative humidity 80%, average yearly rainfall 1288 mm) with moderately acid, loam soils (pH 5.6, Andisol). The field experiment (a randomized complete block design with three replicates and 3 m long single-row plots) was managed with two foliar applications of zinc and boron as microelements (300 g ha^−1^ as chelates) carried out at 14 and 21 days after planting. Harvesting was by hand into clean plastic buckets to avoid soil contamination. Foliar disease pathogens were controlled with the fungicides, mancozeb, and benomyl, at doses of 1.0 and 0.3 kg ha^−1^, respectively.

A sample of the harvest from the first two replicates was carefully placed separately into two paper bags to keep them clean through transport. Back in seed laboratory, the seeds were hand washed with 70% ethanol to remove dust and dried in a stationary oven. The seed were all weighed upon reaching 10% moisture and the seed color was recorded, although most of the genotypes had medium to large red seed (45 to 55 g *per* 100 seed) like the recurrent parent Cerinza. The similarity of the lines was a result of backcrosses of the recurrent cultivated parent as a recipient of the wild donor parent genes.

### Mineral evaluation and data analysis

For each repetition, 12 g of seed were washed with sterile double-distilled water and peeled by hand using a sterile scalpel to remove the seed coat from the cotyledons. The separate seed coat and cotyledon samples were placed in separate envelopes and then dried for 24 h at 45°C in a bench-top oven, before grinding in a modified Retsh mill with 24 sample slots using zirconium grinding balls and Teflon grinding chambers. Seed coat and cotyledonary tissues were dried and weighed out into two replicates of ~0.25 g dry weight each which were analyzed separately before averaging. These samples were analyzed with nitric acid-perchloric acid digestion as described in Blair et al. (2009).

Digested samples were taken to dryness and re-suspended in 15 mL of trace-metal grade nitric acid (2% v/v), prior to multi-element analysis by inductively coupled plasma—optical emission spectroscopy (ICP-OES) (CIROS ICP Model FCE12; Spectro, Kleve, Germany) at the USDA-ARS Children's Nutrition Research Center at the Baylor College of Medicine (Houston TX). The instrument was calibrated daily with certified standards. Tomato leaf standards (SRM 1573A; National Institute of Standards and Technology, Gaithersburg, MD) were digested and analyzed along with the seed samples to ensure accuracy of the instrument's calibration. Because of the large amount of work involved in peeling sufficient seed, only 60 of the full set of 138 AB representing a random selection of lines for full-seed iron concentration were evaluated. These included 20 high, 20 moderate and 20 low seed iron containing lines for the cross covering the full range from 60 to 95 ppm (Table S1) as measured for Darien and Palmira by Blair and Izquierdo (2012). In addition, the two parents, Cerinza and G10022 were used as control genotypes twice in the analysis. Because of the higher seed coat ratio in G10022 only 4.5 g of this wild bean was needed to obtain sufficient seed coat for analysis.

All quantitative data were analyzed using a general linear model and an analysis of variance for a split plot design for genotypes within tissue types analyzed in the software package Statistix v. 8.0 (Analytical Software Inc.) and means were estimated to use for subsequent analysis. Population distributions were evaluated for normality using the same software. Transformations were carried out with natural logarithm in cases were population results were skewed before use for QTL analysis. Pearson's correlations were estimated based on the non-transformed data. QTL analysis was carried out with the means described above and the genetic map built by Blair and Izquierdo (2012). QTL were identified using two software programs QTL Cartographer v. 2.5 (Basten et al., 2001) for composite interval mapping analysis (CIM) and MapDisto v. 1.7 (http://mapdisto.free.fr/) for single point analysis (SPA). In the CIM analysis we performed the analysis every 1 cM (walkspeed) with a window size of 10 cM and using ten background markers in a forward-backward stepwise multiple linear regression model. In terms of population type, the B_12_ genetic model was assumed for the CIM analysis. Meanwhile a simple regression model was assumed for the SPA analysis. In both cases the homozygous donor parent allele class was combined with the heterozygous genotypic class. Significance thresholds were set at LOD 3.0 (*P* ≤ 0.001).

The phenotypic variance controlled by a given QTL was determined by its determination coefficient (R2), as defined by the software program. QTL for micronutrient concentrations were named using the mineral name, the abbreviation for seed coat or cotyledon and a two number code derived from the linkage group and the number of the QTL identified on that linkage group, separated by a period. Genetic maps and QTL locations were drawn with MapChart v. 1.0 software program (Voorips, 2002) where map distances were reported in centiMorgans (cM) estimated with the Kosambi mapping function.

## Results

### Mineral variability in seed coat vs. cotyledons

A total of ten minerals were analyzed by the ICP-OES experiment for the two subsamples namely the seed coat and cotyledon (Table 1). These minerals were boron (B), calcium (Ca), copper (Cu), iron (Fe), potassium (K), magnesium (Mg), manganese (Mn), phosphorus (P), sulfur (S), zinc (Zn). The concentrations were calculated in part per million (ppm) equivalent to g/kg or μg/g. The seed coat was found to have low concentrations compared to the cotyledon for B, Cu, K, Mn and especially P and S. Meanwhile the seed coat had higher concentrations of Ca, Fe, Mg, and Zn.

**Table 1:** **Descriptive statistics for seed coat vs. cotyledonary minerals evaluated as measured by inductively coupled plasma (ICP) analysis for the advanced backcross population of common beans**.

**Seed coat values**	**Minerals evaluated^1^**									
	**B coat**	**Ca coat**	**Cu coat**	**Fe coat**	**K coat**	**Mg coat**	**Mn coat**	**P coat**	**S coat**	**Zn coat**
Minimum (ppm)^2^	7.0	12500	1.0	**20.0**	2000	2000	2.0	325	298	**17.0**
Maximum (ppm)	21.0	21500	3.0	**263.0**	9000	4000	5.5	635	440	**54.0**
Range (ppm)	14.0	9000	2.0	**243.0**	7000	2000	3.5	310	141	**37.5**
Average (ppm)	13.25	16638	1.19	**69.795**	6377	2884	2.684	424	350	**32.428**
SD (ppm)	2.5	1365	0.307	**56.305**	934	265	0.574	56	26	**6.436**
Kurtosis	−0.245	0.217	7.084	**1.532**	1.862	2.281	2.656	0.513	0.340	-**0.018**
Skewing	0.015	0.382	2.670	**1.799**	−0.558	−0.704	1.267	1.048	0.832	**0.582**
CV (%)	23.4	10.8	38.2	**18.4**	19.2	14.0	26.6	16.5	9.3	**25.6**
**Cotyledonary values**	**B coty**	**Ca coty**	**Cu coty**	**Fe coty**	**K coty**	**Mg coty**	**Mn coty**	**P coty**	**S coty**	**Zn coty**
Minimum (ppm)^2^	11.0	189	6.0	**54.5**	12500	1000	12.0	3500	1000	**27.0**
Maximum (ppm)	38.5	374	9.0	**93.0**	15000	1281	21.0	6000	3000	**41.0**
Range (ppm)	27.5	185	3.0	**38.5**	2500	281	9.0	2500	2000	**14.0**
Average (ppm)	15.598	284	7.577	**75.362**	13971	1012	15.576	4332	1982	**32.358**
SD (ppm)	3.028	34	0.751	**5.903**	582	24	1.375	435	79	**2.188**
Kurtosis	10.177	−0.510	−0.763	**0.461**	−1.104	18.066	1.234	0.393	15.181	**0.546**
Skewing	2.868	−0.057	0.105	-**0.553**	−0.064	4.390	0.862	1.074	−1.230	**0.626**
CV (%)	30.5	14.5	11.7	**10.3**	5.2	5.5	11.5	11.6	11.8	**8.8**

1 *Mineral abbreviations: B, boron; Ca, calcium; Cu, copper; Fe, iron; K, potassium; Mg, magnesium; Mn, manganese; P, phosphorus; S, sulfur; Zn, zinc*.

*Tissue Abbreviations: coat, seed coat; coty, cotyledon (and embryo). The minerals of most interest, iron and zinc are indicated in the columns which are in bold text*.

2 *Mineral concentrations given in part per million (ppm); note difference between micronutrients (B, Cu, Fe, Mn, Zn) vs. other minerals (Ca, K, Mg, P and S)*.

For the minerals of greatest interest to our study, average Fe and Zn concentrations of the cotyledon were similar to that of seed coat across the entire population (Table 1). However, the range of Fe in seed coat was very large with a minimum of 20 ppm to a maximum of 263 ppm, while Zn in seed coat ranged from 17 to maximum of 54.5 ppm. By comparison cotyledonary values were less variable ranging from 54.5 to 93 ppm for Fe and 27 to 41 ppm for Zn. For other nutritionally important minerals, Ca ranged from 12500 to 21500 ppm in seed coat which was surprising given average of 284 ppm in cotyledon. The opposite was true for *P* which averaged 4332 in cotyledon but only 424 ppm in seed coat. Similarly S averaged 1982 ppm in cotyledons but only 350 ppm in seed coat. The differences in the concentration averages between seed coat and cotyledon for each of these minerals were significant in each case.

**Figure 1:**
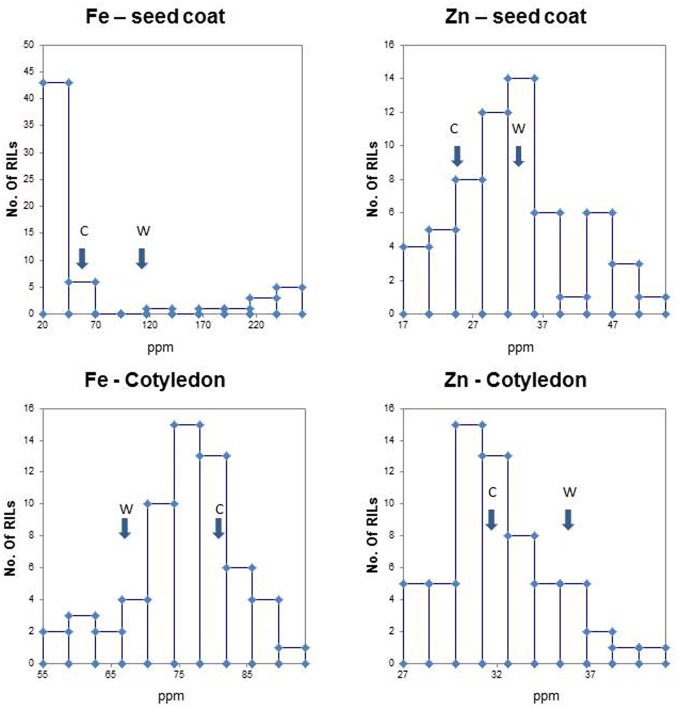
**Population frequency distributions for iron (Fe) and zinc (Zn) concentration in seed coats and cotyledonary tissues in the (Cerinza × (Cerinza × (Cerinza × G10022))) advanced backcross population as determined by ICP analysis**. Arrows indicate phenotypic value of recurrent parent Cerinza (C) and the wild donor parent G10022 (W). The mineral concentration in parts per million (ppm) is found on the x-axis, while the number (no.) of recombinant inbred lines (RILs) is found on the y-axis.

Observation of population distribution types for iron in cotyledons and zinc in both tissues showed normality and a lack of high kurtosis or skewing (Figure 1, Table 1). The exception to this was the binomial distribution and high *K* and *S* values for seed coat iron suggesting simpler inheritance and perhaps a major QTL for this trait. This was in contrast to the normal population distributions for zinc concentrations in seed coat or for iron concentration in the cotyledons. Therefore the inheritance of these minerals in these tissues appears to be controlled by multigenic or quantitative. The other minerals were also graphed for their population distribution (data not shown). The range, median, means and *K* or *S* values all indicate normal distributions and quantitative inheritance for the most part, although the *K* and *S* values were high for seed coat Cu and for cotyledonary B and Mg. Kurtosis was also observed for cotyledonary S (Table 1).

### Statistical associations between minerals

In the ANOVA results, genotype effects for the advanced backcross lines were highly significant (*P* = 0.01–0.001) in each case except B, for which the effect was lower (*P* = 0.05) while tissue effects were either highly or moderately significant (Table 2). Tissue × genotype effects were also highly significant for all minerals. Replication differences were minor as evidenced by low sums of squares values (data not shown), which is explained by the good repeatability of the study, with separate samples managed from the field to the seed room to lab analysis with very similar treatment. Coefficients of variation (CVs) were below 3%, except for B and Ca which had higher CVs for the two error terms used Error (Replication × Tissue) to test significance of Tissue differences and Error (Replication × Tissue × Genotype) to test significance of Genotype and Tissue × Genotype differences (Table 2).

**Table 2:** **Significance in terms of probability (P) of *F*-tests for the analyses of variance for each mineral evaluated by inductively coupled plasma (ICP) analysis for seed coat vs. cotyledonary tissue mineral concentration measured for the advanced backcross genotypes (lines) and parents (Par.) of the advanced backcross population of common beans**.

**Sources^1^**	**Df^2^**	**B-Lines**	**Ca-Lines**	**Cu-Lines**	**Fe-Lines**	**K-Lines**	**Mg-Lines**	**Mn-Lines**	**P-Lines**	**S-Lines**	**Zn-Lines**
Tissues	1	0.0395^*^	0.0057^**^	0.0010^***^	0.0273^*^	0.0024^**^	0.0018^**^	0.0017^**^	0.0008^***^	0.0002^***^	0.0020^**^
Genotypes	59	0.0000^***^	0.0000^***^	0.0000^***^	0.0000^***^	0.0000^***^	0.0000^***^	0.0000^***^	0.0000^***^	0.0000^***^	0.0000^***^
Tissues × Genotypes	59	0.0246^*^	0.0000^***^	0.0000^***^	0.0000^***^	0.0000^***^	0.0000^***^	0.0000^***^	0.0000^***^	0.0000^***^	0.0000^***^
C.V. (%) Rep × Tissue		7.55	13.34	1.58	2.53	2.08	1.70	2.78	1.67	0.39	0.03
CV (%) Rep × Tissue × Genotype		24.89	4.00	2.18	2.89	1.13	2.12	2.30	2.10	2.15	3.31
**Sources^1^**	**Df^2^**	**B-Par**.	**Ca-Par**.	**Cu-Par**.	**Fe-Par**.	**K-Par**.	**Mg-Par**.	**Mn-Par**.	**P-Par**.	**S-Par**.	**Zn-Par**.
Tissues	1	0.0918^ns^	0.0060^**^	0.0007^**^	0.0347^*^	0.0021^**^	0.0067^**^	0.0010^***^	0.0012^**^	0.0035^**^	0.0322^*^
Genotypes	59	0.3790^ns^	0.0275^*^	0.0031^**^	0.0003^**^	0.0013^**^	0.0146^*^	0.0047^**^	0.0022^**^	0.0038^**^	0.0197^*^
Tissues × Genotypes	59	0.0676^ns^	0.0270^*^	0.0711^ns^	0.0001^***^	0.0185^*^	0.0022^**^	0.0020^**^	0.0037^**^	0.0077^**^	0.1736 ^ns^
C.V. (%) Rep × Tissue		3.25	2.59	0.21	0.76	0.28	0.93	0.33	0.47	1.17	1.52
CV (%) Rep × Tissue × Genotype		7.14	5.86	2.92	0.67	1.08	1.40	1.21	1.55	2.74	2.03

1 *Mineral abbreviations for sources of variation: B, boron; Ca, calcium; Cu, copper; Fe, iron; K, potassium; Mg, magnesium; Mn, manganese; P, phosphorus; S, sulfur; Zn, zinc*.

Tissue Abbreviations: coat, seed coat; coty, cotyledon (and embryo). Significance codes:

*** *= 0.001*,

** *= 0.01*,

* *= 0.05, ns, not significant*.

2 *Df, Degrees of freedom, based on the ANOVA analysis*.

Parental comparisons showed that for the most part tissue differences were highly to moderately significant as were genotype differences, except in the case of the first mineral, B (*P*> 0.05), which was non-significant for any of the sources of variation (Table 2). Tissue × genotype effects were also non-significant for Cu and Zn amongst the parents but were significant for all other minerals although to a lesser extent and lower probability than for the advanced backcross lines.

Similarly, probability values for differences between the tissues were less significant than for the lines in most cases although this was more notable for the minerals B and Fe among the lines (differences at *P*≤ 0.05) with all other minerals surpassing the level of high significance (differences at *P*≤ 0.001). Some of the genotype comparisons for minerals were very highly significant (differences at *P*≤ 0.0000). Tissue × genotype effects were less observable for B but were also of very high significance for the remainder of the minerals (differences at *P*≤ 0.0000).

The average parental values for Fe and Zn (Figure 1, arrows) were of interest given our attention to these micronutrients. Most noticeably, the wild donor parent (G10022) had more than double the seed coat Fe concentration (93.28 ppm) of the cultivated recurrent parent (Cerinza, 40.52 ppm). Meanwhile the cotyledonary Fe concentrations were similar but reversed with Cerinza having higher iron in the cotyledons (80.16 ppm) than the cotyledons of G10022 (67.51 ppm). Differences were significant based on paired *t*-tests, but higher for seed coats (*P*= 0.0039) than for cotyledons (*P*= 0.0180). For Zn concentrations the cotyledons were about 6–8 ppm richer in this mineral than the seed coats. However, in both cases G10022 had more Zn (28.7 ppm in seed coat, 36.2 ppm in cotyledons) than Cerinza (26.5 ppm in seed coat, 32.16 ppm in cotyledons). Paired *t*-tests showed high significance for the contrast in seed coat (*P*= 0.0033) but non-significance for the contrast in the cotyledon (*P*= 0.0684).

The Pearson's correlation coefficient results (Table 3) confirmed tissue × genotype interaction and that the relationships among minerals was different in the two types of tissues, the seed coat and the cotyledon. For cotyledonary tissue, Mn was correlated with P and S just as K was correlated with Mn but none of the negative correlations were highly significant. Less significant (*P*≤ 0.05) and negative correlations of Ca and K or Fe and Mg were observed in the cotyledons. Likewise positive correlations (*P*≤ 0.01) for Cu and K or Mg and Mn were observed. It was surprising that Fe was not correlated with P or Zn in the cotyledon.

**Table 3:**
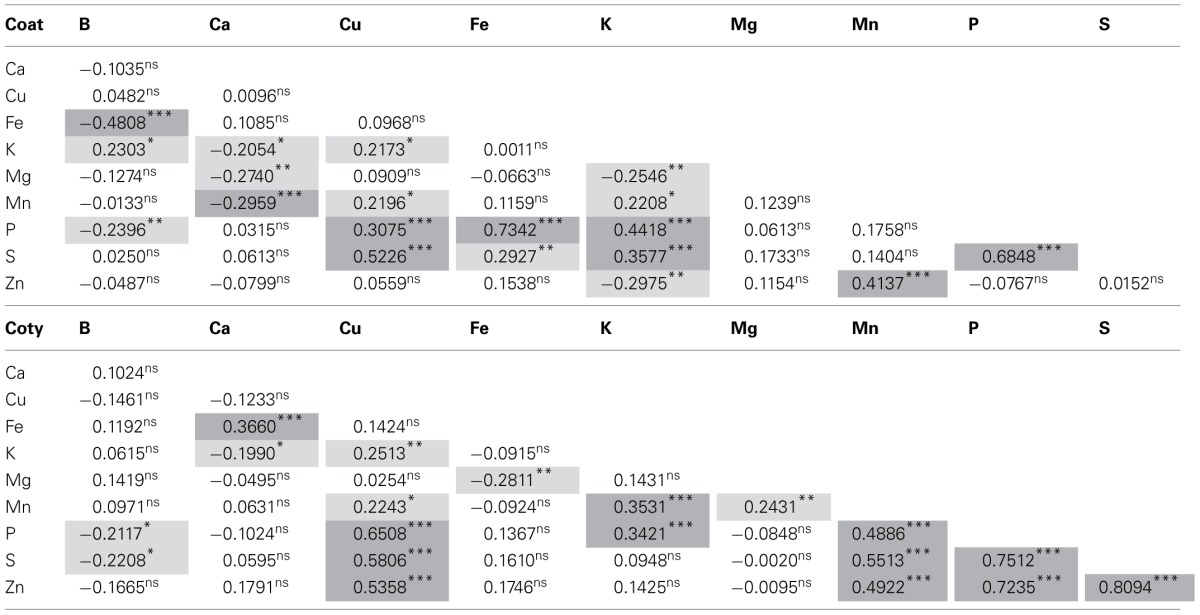
**Pearsons correlation coefficients and significance for ten minerals^1^evaluated by inductively coupled plasma (ICP) analysis for seed coat and cotyledonary tissue in the advanced backcross population of common beans**.

*^1^Mineral abbreviations for correlation coefficients: B, boron; Ca, calcium; Cu, copper; Fe, iron; K, potassium; Mg, magnesium; Mn, manganese; P, phosphorus; S, sulfur; Zn, zinc*.

Tissue Abbreviations: coat, seed coat; coty, cotyledon (and embryo). Significance codes: ^***^= 0.001, ^**^= 0.01, ^*^= 0.05, ns, not significant, as indicated by dark to light shaded boxes, with no significance non-shaded.

Meanwhile, for seed coat tissues Fe was positively correlated with P and S (at *P*≤ 0.01 and *P*≤ 0.001, respectively) but not with Zn. Potassium and Cu levels were positively and highly correlated (*P*≤ 0.001) with those of *P*and *S*but less so with Mn (*P*≤ 0.01). Highly significant negative associations existed between Fe and B as well as between Mn and Ca in the seed coat. Ca was also negatively correlated with K and Mg in seed coat but at lower significance (*P*= 0.01 and *P*= 0.05, respectively).

### Inheritance of seed coat and cotyledonary iron and zinc

**Figure 2:**
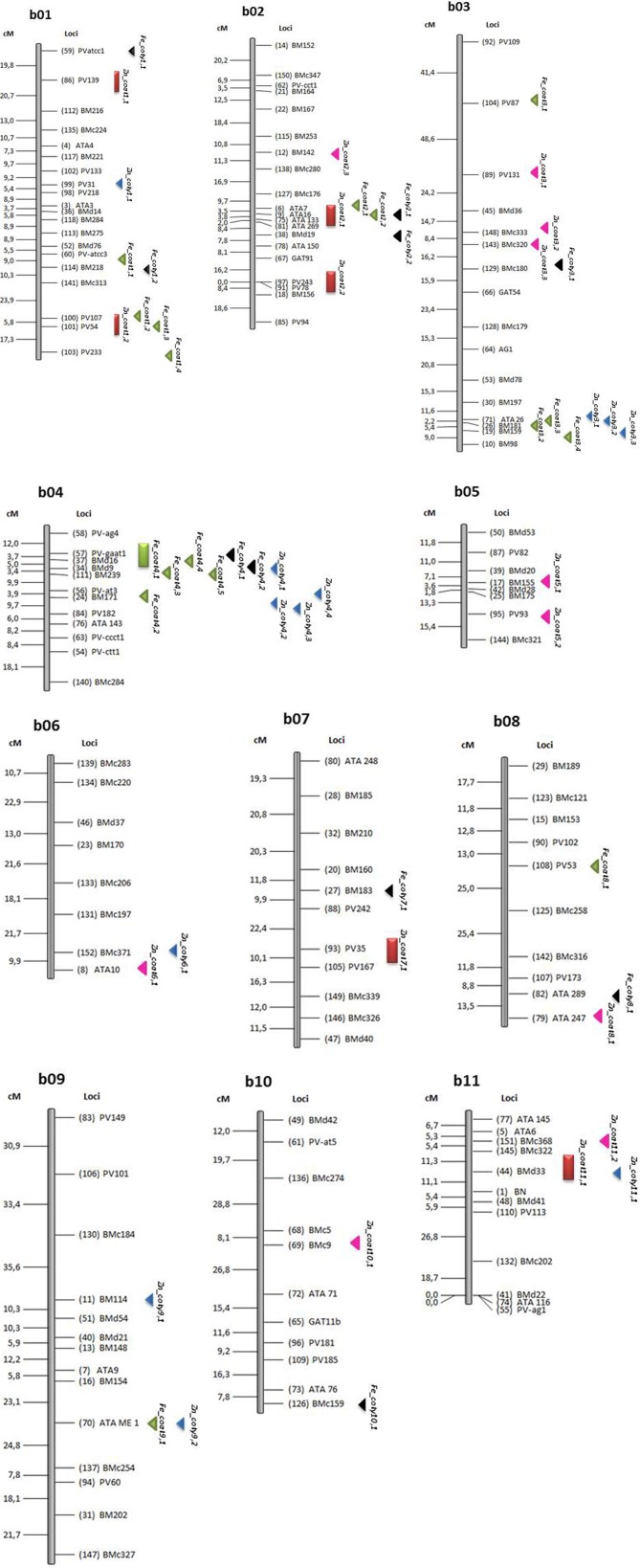
**Location of quantitative trait loci (QTL) for iron (Fe) and zinc (Zn) concentration in seed coat**. Those QTL identified by CIM analysis in the (Cerinza × (Cerinza × (Cerinza × G10033))) advanced backcross population are indicated by thick bars acompanied by a QTL name as indicated in Table 4. QTL identified for Fe and Zn with SPA analysis as indicated in Table 5are shown as left pointing arrow heads pointing towards the most significant markers. Abbreviations for QTL are based on Fe, iron; Zn, zinc in seed coat; coat or cotyledon, coty.

Using only the Fe and Zn datasets, a total of 7 QTL were found with CIM analysis and permutations for significance threshold (Table 4and Figure 2) and 49 for significance at *P*= 0.05 with SPA analysis (Table 4and Figure 2). Among the QTL identified with CIM all were for seed coat Fe and Zn and none were found for cotyledonary Fe and Zn. The seed coat CIM—QTL were divided into six Zn loci on linkage groups B01, B02, B07, and B11 and one Fe locus on linkage group B04 based on linkage group assignments. LR values ranged from 9.49 for the Fe QTL to 29.53 for one of the Zn QTL. It was notable that the Fe QTL and three of the Zn QTL, including one with the second highest LR value, were derived from the high mineral wild donor parent, G10022. Three other QTL for Zn concentration, including one with the highest LR values and one with the third highest LR value were from the cultivated recurrent parent, Cerinza. Two linkage groups contained more than one QTL for Zn concentration but in each case the two QTL were derived from the same parental allele. For example on linkage group B01, both QTL had positive alleles from G10022; while on B02, both QTL had positive alleles from Cerinza. The additive effect of the Fe QTL showed that this represents a major gene that can provide up to 110 ppm increased seed coat mineral concentration, which would change the balance and total concentration of Fe in the whole seed. The additive effects of the Zn QTL were smaller, ranging from 3.6 to 12.1 ppm increases which are also important in total seed mineral concentration considering the range of variability for Fe (243 ppm) and Zn (32.43) in seed coat. For example the additive effect of 12.1 ppm for Zn is 44.53% increase and very similar to that observed for Fe (45.27%).

**Table 4:** **Quantitative trait loci for seed coat iron and zinc concentration identified by composite interval (CIM) mapping analysis in the advanced backcross population derived from the wild donor parent (G10022) and the recurrent cultivated parent (Cerinza)**.

**Trait**	**QTL name**	**Chr**.	**Position**	**LR**^1^	**Additivity**	**Source**	**R2**	**Nearest marker^2^**
**SEED COAT QTL**								
Fe		4	0.0901	9.49	109.8173	G10022	0.087679	PV-gaat1
Zn		1	0.1701	27.93	5.7301	G10022	0.062669	PV139
Zn		1	1.3701	12.41	3.6300	G10022	0.035964	PV54
Zn		2	2.4201	22.69	4.9477	Cerinza	0.042576	ATA133
Zn		2	3.3001	29.53	5.4577	Cerinza	0.042892	PV78
Zn		7	2.4801	13.95	12.1286	Cerinza	0.274638	PV35
Zn		11	0.6001	13.83	5.4419	G10022	0.077907	BMd33
**COTYLEDON QTL**								
Fe	None found	0	NA	NA	NA	NA	NA	NA
Zn	None found	0	NA	NA	NA	NA	NA	NA

1 *LR, likelihood ratio test statistic for H_0_:H_1_where H_0_is the hypothesis of no QTL effect at test position and H_1_is the hypothesis of a QTL effect at the test position; R2, proportion of variance explained by the QTL at test site; Values have significance at 0.5% probability after 1000-fold permutation tests*.

2 *The nearest marker is the marker closest to the peak LR score*.

Although many SPA—QTL were identified for iron and zinc (Table 5) these results agreed with the results from CIM—QTL analysis in terms of major locations and source of high iron or zinc alleles, validating the previous analysis. SPA results for minerals other than Fe and Zn are shown in Table S2. Of the biofortification SPA - QTL, a total of 16 were for Fe in the seed coat and 10 were for Fe in the cotyledon. All of the seed coat Fe QTL were the result of alleles from G10022, the very high seed coat iron parent, and four of the associated markers with highest additive effect were on B04. The phenotypic variance explained by each of the markers ranged from 7.2 to 25.3 % with the marker Pv-at03 and the surrounding markers on linkage group B04 being the most important, confirming the results of a major locus detected with CIM analysis. The additive effects of substitution of the G10022 allele for any of these markers was an amazing 113 to 171 ppm of iron with BMd9 the most significant marker in terms of additivity.

**Table 5:** **Quantitative trait loci for seed coat or cotylendon iron (Fe) and zinc (Zn) concentration identified by single point analysis (SPA) in the advanced backcross population derived from the wild donor parent (G10022) and the recurrent cultivated parent (Cerinza)**.

**LG**	**Marker**	**Significance**			**Additivity**	**Source**
		***F***	***R*^2^**	***P***		
**Fe COAT**						
1	BM218	5.23	0.083	0.026	79.16	G10022
1	PV107	5.63	0.094	0.021	57.80	G10022
1	PV54	5.06	0.090	0.029	59.94	G10022
1	PV233	6.81	0.118	0.012	81.59	G10022
2	BMc176	7.51	0.128	0.008	82.73	G10022
2	ATA16	5.55	0.090	0.022	172.43	G10022
3	PV87	6.19	0.100	0.016	54.30	G10022
3	ATA 26	5.05	0.101	0.029	100.33	G10022
3	BM159	4.12	0.070	0.047	58.88	G10022
3	BM98	4.96	0.083	0.030	54.60	G10022
4	Pv-at3	17.36	0.258	0.000	127.50	G10022
4	PV182	10.59	0.172	0.002	113.25	G10022
4	BMd16	6.32	0.098	0.015	132.87	G10022
4	BMd9	5.26	0.083	0.025	171.37	G10022
8	PV53	4.17	0.072	0.046	73.31	G10022
9	ATA ME1	5.03	0.093	0.029	74.97	G10022
**Fe COTYLEDON**						
1	PVatcc1	4.74	0.076	0.033	6.61	Cerinza
1	BMc313	6.15	0.116	0.017	8.54	Cerinza
2	ATA7	4.22	0.069	0.044	15.78	Cerinza
2	GAT91	6.36	0.104	0.015	9.58	G10022
3	BMc180	4.02	0.072	0.050	7.15	G10022
4	PV-ag4	4.95	0.084	0.030	7.85	Cerinza
4	BMd16	5.27	0.083	0.025	12.42	Cerinza
7	BM183	8.82	0.143	0.004	14.44	Cerinza
8	ATA 289	7.64	0.128	0.008	21.08	Cerinza
10	BMc159	9.23	0.151	0.004	21.89	Cerinza
**Zn COAT**						
2	BM142	5.03	0.088	0.029	7.43	Cerinza
3	PV131	4.57	0.089	0.038	6.40	G10022
3	BMc333	4.98	0.092	0.030	10.84	G10022
3	BMc320	4.74	0.079	0.034	10.34	G10022
5	BMd20	4.59	0.073	0.036	10.34	G10022
5	PV93	5.60	0.096	0.021	8.44	G10022
6	ATA10	7.95	0.124	0.007	10.62	G10022
8	ATA 247	13.07	0.246	0.001	12.73	G10022
10	BMc9	4.26	0.074	0.044	6.75	G10022
11	BMc368	4.98	0.082	0.030	17.09	G10022
**Zn COTYLEDON**						
1	PV133	5.53	0.091	0.022	3.12	Cerinza
3	ATA 26	15.75	0.259	0.000	5.66	G10022
3	BM159	4.93	0.082	0.030	2.48	G10022
3	BM98	6.33	0.103	0.015	2.29	G10022
4	BMd9	11.36	0.164	0.001	9.03	G10022
4	PV182	9.94	0.163	0.003	4.43	G10022
4	ATA 143	8.10	0.135	0.006	3.47	G10022
4	BM171	5.85	0.098	0.019	4.93	Cerinza
6	BMc371	4.94	0.088	0.031	2.96	Cerinza
9	ATA ME 1	10.37	0.175	0.002	3.58	G10022
9	BM114	4.22	0.081	0.045	2.51	G10022
11	BMd33	4.64	0.077	0.035	3.17	Cerinza

Other slightly less important SPA-QTL seed coat Fe were detected on B02 (at ATA16) and B03 (at ATA26), but these still had major additive effects of up to 172 and 100 ppm iron concentration, respectively, for the best marker in each region. The phenotypic effects explained by these regions averaged 10 and 9%, respectively. A more minor QTL was found on B01 with smaller additive effects but still contributing over 55 ppm which is more than the average seed concentration of most common beans. The phenotypic variances explained by these markers (PV54, 107, and 233) were around 10%.

Iron in the cotyledon presented a total slightly smaller number of QTL (10) than seed coat QTL (16) with the SPA method. However, in contrast to seed coat QTL, most of these SPA-QTL for cotyledonary Fe (8) were derived from Cerinza and only 2 were from G10022. The position of the QTL for cotyledon Fe were different than those for the seed coat Fe on some linkage groups for example B07 and B10. On the other hand seed coat and cotyledon QTL overlapped on some linkage groups (B01, B02, B03, B04, and B08). On linkage group B04, in both cases QTL were linked to BMd16 marker for seed coat and cotyledon, although from a different source (Cerinza instead of G10022, respectively). The effects of these QTL were significant as seen in the additivity values of 6.6 to 21.9 ppm (up to 56.9% more iron) considering the range of variability for cotyledon Fe (38.5 ppm). The QTL explained 6.9–15.1% of phenotypic variance.

In terms of Zn QTL, the SPA method detected 10 QTL for seed coat concentration and 12 QTL for cotyledon concentration. Among the first of these QTL the associated markers were distributed across linkage groups B02, B03, B05, B06, B08, B10, and B11 with most of the high Zn alleles (9) coming from G10022, except for the first one on B02. Their importance however in terms of additivity was more minor (6.4–12.7 ppm) although the explained variance ranged from 7.3 to a high of 24.6%. In the case of the cotyledonary zinc SPA-QTL, 4 were derived from Cerinza and 8 from G10022 and their additive effect ranged from 2.3 to 9.0 ppm zinc and were found on B01, B03, B04, B06, B09, and B11. In terms of overlap, the Zn SPA-QTL on linkage group B02 were different than the CIM-QTL identified on this same linkage group. In summary some seed coat Fe or Zn were in similar locations as cotyledonary QTL while others were in different locations than for cotyledonary QTL.

## Discussion

The major results of this study present a quandary for biofortification breeding: namely, that both the distribution and the inheritance of micronutrients is different in the maternally derived seed coat tissue versus in the cotyledonary tissues which are part of the embryo which will germinate in the next sporophytic generation. These results confirm the variability in seed coat Fe found by Ariza-Nieto et al. (2007) and Moraghan et al. (2002): however, our genetic results are new and differ from previous inheritance studies that evaluated micronutrient concentrations in whole seed in other populations (Blair et al., 2009, 2010a, 2011) or with the same population (Blair and Izquierdo, 2012). When dealing with maternally-derived seed coat tissue we must think about the seed-producing generation in terms of genetics, plant growth and seed development. For any seed trait the previous season's growing conditions are important too.

The importance of differences in micronutrient distribution in seed coat and the rest of the seed resides in the interaction of minerals between each other and with secondary metabolites such as phytates or tannins that vary in concentration as well as between seed coat and cotyledonary tissues (Moraghan, 2004; Ariza-Nieto et al., 2007). In terms of inheritance of the micronutrient accumulation traits, the importance of a difference in seed coat and cotyledon resides in the presumably different genes involved in each tissue's mineral accumulation. It was not surprising therefore that we found a different set of QTL for seed coat Fe and Zn in this study compared to our previous study working with whole seed (Blair and Izquierdo, 2012), especially in terms of the major CIM-QTL. Seed coat mineral accumulation is important to consider for breeding of micronutrient rich beans due to the bioavailability of micronutrients of this portion of the seed (Ariza-Nieto et al., 2007) and the GxE effects that are found to affect mineral accumulation (Blair et al., 2010b).

The genetic results described above showed that most minerals were normally distributed both in seed coat and cotyledon or at least in cotyledon across the population. These data proved the multi-genic or quantitative inheritance of mineral concentrations. One exception was the set of values for Fe in seed coat, which had a bimodal distribution indicating a major CIM-QTL and qualitative inheritance which was found to be on linkage group B04. This was in contrast to seed coat Zn concentration which was normally distributed and had six CIM-QTL, which were found on four different linkage groups. SPA–QTL were more abundant and will be discussed as well.

Blair and Izquierdo (2012), with the same population but analysis of whole seed, found the same CIM based QTL for Zn accumulation on B07 near marker PV35 but different QTL for Fe concentration or content on B07 and B08, respectively. Some SPA based QTL for Fe and for Zn from Blair and Izquierdo (2012) overlapped with the seed coat or cotyledon based SPA - QTL on B01, B04, B10, and B11.

The QTL for zinc concentration on B11 were especially important in another Andean × Mesoamerican cross study (Blair et al., 2009). The QTL for zinc concentration on B01 near PV139 may have been influenced by the *fin*locus as was found by Cichy et al. (2009) for another cross involving a determinate Andean bean, similar in growth habit to Cerinza. The *fin*locus was shown to control the determinacy but not the height of bean plant growing shoots (Chavarro and Blair, 2010) and in the advanced backcross population analyzed here was observed to affect total biomass production and plant size (Izquierdo et al., unpublished results). One possible hypothesis could be that larger indeterminate *Fin Fin*plants accumulated a large amount of zinc in vegetative tissue and this zinc was available for translocation to the seed. Meanwhile, the shorter and smaller biomass *fin fin*plants that are determinate in growth habit would accumulate less zinc in both vegetative and reproductive tissues. A different Zn QTL found on linkage group B02 near PV78 and derived from Cerinza was in close proximity to the QTL *ZnPoAAS2.1*found by Blair et al. (2010a) in a Mesoamerican x Mesoamerican cross and another QTL *Zn-AAS2c*found by Blair et al. (2011) in an Andean × Andean cross.

On the other hand, many additional SPA-QTL from this study appear to be novel based on their evaluation in the different tissues. For example, the seed coat Fe QTL on B04 derived from G10022 was not detected near any Fe concentration QTL in the cultivated Andean × Andean or Mesoamerican × Mesoamerican populations studied by (Blair et al., 2010a, 2011) and may be specific to wild bean sources. This major QTL from linkage group B04 probably influenced the observation of binomial population distribution in the population for seed coat iron concentration.

The lack of cotyledonary CIM-QTL for iron or zinc in the present study may have been a reflection of the smaller differences between parents for the Fe and Zn concentrations in this tissue, although SPA-QTL analysis identified a good number of markers with significant effects. This may show that epistatic interactions are sometimes important in identification of QTL for the minerals due to the dependence of iron translocation to the seed on the amount of iron uptake by the roots into the plant. More QTLs were found with the SPA method due to the lower probability threshold (*P*≤ 0.05) used in that analysis compared to the CIM method where thresholds were determined with permutations. The lack of cotyledonary CIM iron QTL was a result of high thresholds found for this variable's analysis. Despite this, the cotyledonary QTL where in similar locations to QTL found in the whole seed for the same population by Blair and Izquierdo (2012).

From a breeding perspective, the lines with high seed coat Fe concentration might be of interest if this Fe is shown to be bioavailable although initial results suggest that seed coat iron especially in colored beans is not very bioavailable (Ariza-Nieto et al., 2007). It was notable that some lines of the population had seed coat Fe concentrations of more than 250 ppm, while seed coat Zn concentrations above 50 ppm might be of interest. The microsatellite that were associated with QTL for seed coat mineral concentration could be used for marker assisted selection (MAS) of these traits as was suggested for whole seed concentration QTL by Blair and Izquierdo (2012).

Considering that the seed coat makes up approximately 10% of the seed the amount of iron in the seed coat can have a large impact on the amount of iron in the entire seed. In populations like the advanced backcross lines, the amount of seed coat is fairly uniform but the amount of seed coat Fe was not. In a typical recombinant inbred line population that segregates for seed size, the percentage seed coat, the ratio of seed coat to total seed and the amount of seed coat Fe would all be variables to study. The result of the high iron concentration in the seed coat and the high percentage of seed coat to seed weight is that seed coat Fe at high concentrations affects the overall average Fe concentration of the seed quite substantially. For example, from this study and our previous results (Blair and Izquierdo, 2012), the total amount of seed iron can increase or decrease by up to 40 ppm based mostly on seed coat Fe content.

The results we observed for correlation among minerals for cotyledons are different than Fe-Zn relationships observed previously for whole seed in several populations (Blair et al., 2009, 2010a, 2011) and in the same advanced backcross population (Blair and Izquierdo, 2012). Meanwhile seed coat results showed interactions of Fe and P which are new information for seed coat, but there was no correlation for Fe and Zn, in contrast to Blair et al. (2009). A relationship of Fe and P in seed coat was surprising considering that phytate which binds Fe should not be found in high concentrations in the seed coat but rather in the cotyledons (Ariza-Nieto et al., 2007).

The Fe and P correlation in seed coat but not in cotyledons can be influenced by the fact that concentrations of P in the seed coat are only one-tenth that of P concentrations in the cotyledons and that in seed coat P may be binding with tannins and other seed coat substances that also bind Fe (Blair et al., 2012). The lack of correlation in the cotyledon between P and Fe might be because Fe in legumes is targeted to vascular cells where starch also accumulates in amyloplast and is found bound to ferritin (Cvitanich et al., 2010). Perhaps phytates are not important in sequestering iron in the seed but rather only as anti-nutrients in the human digestive system (Ariza-Nieto et al., 2007). On the other hand P fertilization is known to affect Zn uptake by plants (Moraghan and Grafton, 2002) and Zn QTL detection (Cichy et al., 2009). The number of Zn QTL contrasts with single gene inheritance found by Cichy et al. (2005) in navy beans, but agrees with multiple gene inheritance found in later studies (Blair et al., 2009, 2010a, 2011; Cichy et al., 2009).

Our hypothesis of a major gene for Fe in seed coat on B04 is the first time a single gene has been proposed for control of this mineral's accumulation in the seed coat of common bean and therefore might be a target for gene cloning and characterization. It would be interesting to know if the seed coat accumulation of Fe is based on a seed coat expressed gene or a gene that diminishes loading of iron into the seed's embryo/cotyledons. Based on co-localization or the lack thereof, ferritin is unlikely to be the mechanism of iron accumulation in seed coat but binding with seed coat tannins could be possible (Ariza-Nieto et al., 2007; Cvitanich et al., 2010). The location of the QTL on the long arm of B04 is interesting as nearby genes for phytohemaglutinnins are expressed only in seeds (Blair et al., 2010c). The clustering of genes expressed in the same tissue is often typical.

Whatever the case, the specificity of the gene to the wild bean source is interesting and could be relevant for gene discovery in other legumes, such as the model species *Medicago truncatula*, where transcriptome analysis has shown the large number of genes (over 30,000) expressed in the seed coat (Verdier et al., 2013). Before this study, the high iron of wild beans could be questioned as an artifact based on the high ratio of seed coat to total seed size in these very small seeded seeds (less than 10 g per 100 seed). If this is the case it would be difficult to use wild beans as a source of high iron. However, it appears that wild beans might preferentially accumulate iron in their seed coats compared to cultivars. Alternatively, cultivars might have been selected to accumulate less iron in their seed coats than wild beans as part of domestication and development of large seed sized domesticates. Selection processes may have been different in large-seeded Andean vs. small-seeded Mesoamerican beans or in very thick-seed coated species such as scarlet runner bean and year-long bean (Singh, 2001).

The idea that human selection for these invisible micronutrient traits has been active is intriguing. We could postulate that this selection was conducted through proxy mechanisms such as seed coat color, palatability, cooking time or noticeable health benefits. Seed coat color is an obvious trait that differs greatly in common bean cultivars with some of these colors associated with tannin or anthocyanin accumulation (Caldas and Blair, 2009; Díaz et al., 2010). Perhaps humans instinctively were attracted to low tannin varieties of white and yellow beans in some cultures or developed methods to remove tannin in red and black beans by soaking, thus also affecting palatability.

Palatability is hard to measure, but cooking time parameters have been evaluated in various studies that also have looked at micronutrient concentration. For example, Saha et al. (2009) found that zinc concentration was negatively correlated with swelling and hydration capacity as components of cooking quality. A few high iron genotypes from that study were also hard to cook. The amount of Fe in the seed coat might have implications for the cooking method recommended when aiming to preserve the nutritional quality of common beans (Ariza-Nieto et al., 2007; Carvalho et al., 2012). As discussed, some of the QTL specific to the seed coat concentration for Fe and Zn were the same as those identified for overall mineral content and might lead Fe to be more likely to stay in the seed when cooked.

The idea that farmer-consumers selected for micronutrient density in the parts of the seed that were more bioavailable in their cultivars compared to their wild relatives is not far-fetched: iron consumption may be noticeable in a feeling of more energy from avoiding anemic status which would have been connected to the foods eaten. The diet of Amerindians from the tropical New World who domesticated common beans was unlike the diets of Indigenous people to the north or south which were very low in animal protein sources. Therefore, the traditional diet of pre-Colombian Middle American and Andean cultures are almost exclusive in vegetable protein sources with basic staples such as maize, beans and squash that were better in amino acids, micronutrients and vitamins than the staples of “Old World” consumers, who had carbohydrate-rich millets, sorghum, rice and wheat. Beans are known as the “meat of the poor.” Perhaps they should be better known as “meat of the Americas” and as “a substitute for animal protein” as many poor people or vegetarians in Africa, Asia and the Americas are well aware of, based on their high consumption of beans.

In conclusion, further molecular and physiological approaches are needed to explain the mechanism for iron accumulation in the seed coat versus the cotyledons but this study provides a starting point for gene and tissue analysis of seed coat minerals. Additional work should look into why there is a very different genetic control of seed iron and zinc tissue distribution in wild vs. cultivated beans, with higher concentrations of iron accumulating in the seed coat of wild beans, but lower concentrations in their cotyledons, and the reverse being the case for cultivated beans. In addition to a major gene on B04 for seed coat Fe in both CIM and SPA analysis, it was notable that the most important of the seed coat Zn SPA-QTL was from a region of the genome on linkage group B11, that has been important in other studies (Blair et al., 2009; Cichy et al., 2009) and that contains ZIP type transporters, which have a role in the uptake and transport of iron in plants (K. Cichy, pers. Communic). Some other regions on B01, B02, B03, B04, B09, and B10 overlap for QTL with those of studies of Fe and Zn accumulation in whole beans (Blair et al., 2009, 2010a, 2011; Cichy et al., 2009). The distribution of QTL for other minerals or the effect of seed weight QTL on B02, B03, and B09 might be worth looking at in more detail in respect to Fe and Zn accumulation. The implications of all this work for biofortification of beans should all be considered in molecular breeding and physiological analysis. Genetic studies along with candidate gene analysis provide the framework to understand the physiology of iron uptake into the plants, transfer to the pod and seed and accumulation in different seed tissues.

### Conflict of interest statement

The authors declare that the research was conducted in the absence of any commercial or financial relationships that could be construed as a potential conflict of interest.
