# Supplementary material for: A legume biofortification quandary: variability and genetic control of seed coat micronutrient accumulation in common beans
Source: Front Plant Sci. 2013 Jul 29;4:275. doi: 10.3389/fpls.2013.00275 (PMC3725406; doi:10.3389/fpls.2013.00275)
Supplement: Supplementary file 1 [file 50458_Blair_DataSheet1.DOCX]

**Supplementary Table 1.** Quantitative trait loci for seed coat or cotylendon concentration of eight minerals identified by single point analysis (SPA) in the advanced backcross population derived from the wild donor parent (G10022) and the recurrent cultivated parent (Cerinza).

| **Mineral** | **LG** | **Marker** | **Significance** | | | | | **Additivity** | **Source** |
| --- | --- | --- | --- | --- | --- | --- | --- | --- | --- |
|  |  |  | **F** | | **R2** | | **P** |  |  |
| **B coat** |  |  |  | |  | |  |  |  |
|  | 2 | ATA16 | 4.29 | | 0.071 | | 0.043 | 6.34 | G10022 |
|  | 3 | BM159 | 4.14 | | 0.070 | | 0.047 | 2.46 | G10022 |
|  | 3 | BM98 | 6.45 | | 0.105 | | 0.014 | 2.47 | G10022 |
|  | 4 | PV-at3 | 5.42 | | 0.098 | | 0.024 | 3.33 | G10022 |
|  | 6 | BMc371 | 4.52 | | 0.081 | | 0.038 | 3.08 | Cerinza |
|  | 7 | PV35 | 4.27 | | 0.079 | | 0.044 | 2.63 | G10022 |
|  | 8 | PV102 | 4.41 | | 0.072 | | 0.040 | 2.54 | G10022 |
|  |  |  |  | |  | |  |  |  |
| **B cotyledon** |  |  |  | |  | |  |  |  |
|  | 1 | PV133 | 7.20 | | 0.116 | | 0.010 | 5.81 | Cerinza |
|  | 4 | BM171 | 9.61 | | 0.151 | | 0.003 | 10.07 | Cerinza |
|  | 1 | PV233 | 6.77 | | 0.117 | | 0.012 | 5.36 | Cerinza |
|  | 3 | BMc180 | 4.66 | | 0.082 | | 0.035 | 4.84 | Cerinza |
|  | 6 | BMc371 | 4.08 | | 0.074 | | 0.048 | 4.60 | Cerinza |
|  | 6 | ATA10 | 5.61 | | 0.091 | | 0.021 | 5.15 | Cerinza |
|  | 7 | ATA 248 | 4.32 | | 0.084 | | 0.043 | 5.46 | Cerinza |
|  | 9 | BM202 | 4.17 | | 0.080 | | 0.046 | 5.15 | Cerinza |
|  |  |  |  | |  | |  |  |  |
| **Ca coat** |  |  |  | |  | |  |  |  |
|  | 6 | BMc220 | 8.64 | | 0.134 | | 0.005 | 1870 | G10022 |
|  | 10 | PV-at5 | 9.98 | | 0.238 | | 0.003 | 3240 | G10022 |
|  | 1 | PV133 | 5.60 | | 0.092 | | 0.021 | 1800 | G10022 |
|  | 4 | BM171 | 4.49 | | 0.077 | | 0.039 | 2510 | G10022 |
|  | 6 | BM170 | 6.40 | | 0.104 | | 0.014 | 1910 | G10022 |
|  | 8 | BMc258 | 6.06 | | 0.104 | | 0.017 | 1600 | G10022 |
|  | 11 | BMc368 | 6.34 | | 0.102 | | 0.015 | 3970 | G10022 |
|  | 11 | BMd41 | 4.93 | | 0.080 | | 0.030 | 1860 | G10022 |
|  |  |  |  | |  | |  |  |  |
| **Ca cotyledon** | |  |  | |  | |  |  |  |
|  | 5 | BMd28 | 8.92 | | 0.140 | | 0.004 | 800 | Cerinza |
|  | 6 | BMc220 | 7.12 | | 0.113 | | 0.010 | 400 | G10022 |
|  | 1 | BMc313 | 4.77 | | 0.092 | | 0.034 | 400 | G10022 |
|  | 2 | BM167 | 6.39 | | 0.102 | | 0.014 | 400 | Cerinza |
|  | 3 | BMc180 | 4.47 | | 0.079 | | 0.039 | 400 | Cerinza |
|  | 4 | PV-ag4 | 6.01 | | 0.100 | | 0.017 | 500 | G10022 |
|  | 5 | BM155 | 5.41 | | 0.090 | | 0.024 | 500 | Cerinza |
|  | 5 | BM175 | 4.30 | | 0.072 | | 0.043 | 500 | Cerinza |
|  | 7 | BM183 | 5.42 | | 0.093 | | 0.024 | 700 | G10022 |
|  | 7 | BMc326 | 5.21 | | 0.088 | | 0.026 | 900 | Cerinza |
|  | 8 | BM153 | 5.42 | | 0.090 | | 0.024 | 400 | Cerinza |
|  | 11 | BMc368 | 5.56 | | 0.090 | | 0.022 | 1000 | G10022 |
|  |  |  |  | |  | |  |  |  |
| **Cu coat** |  |  |  | |  | |  |  |  |
|  | 8 | ATA 289 | 19.93 | | 0.277 | | 0.000 | 1.62 | Cerinza |
|  | 9 | BM202 | 15.38 | | 0.243 | | 0.000 | 0.81 | Cerinza |
|  | 10 | BMc159 | 12.30 | | 0.191 | | 0.001 | 1.60 | Cerinza |
|  | 4 | PV-ag4 | 11.33 | | 0.173 | | 0.001 | 0.72 | Cerinza |
|  | 5 | BMd20 | 7.92 | | 0.120 | | 0.007 | 0.77 | Cerinza |
|  | 11 | ATA6 | 9.93 | | 0.207 | | 0.003 | 1.10 | Cerinza |
|  | 1 | BMc313 | 4.72 | | 0.091 | | 0.035 | 0.48 | Cerinza |
|  | 3 | BM197 | 4.38 | | 0.078 | | 0.041 | 0.37 | Cerinza |
|  | 9 | PV149 | 5.10 | | 0.098 | | 0.028 | 0.42 | Cerinza |
|  | 10 | BMc5 | 5.37 | | 0.095 | | 0.024 | 0.33 | G10022 |
|  | 10 | GAT11b | 5.69 | | 0.100 | | 0.021 | 0.82 | Cerinza |
|  |  |  |  | |  | |  |  |  |
| **Cu cotyledon** | |  |  | |  | |  |  |  |
|  | 1 | PV133 | 7.57 | | 0.121 | | 0.008 | 1.01 | G10022 |
|  | 4 | BM171 | 10.03 | | 0.157 | | 0.002 | 1.73 | G10022 |
|  | 1 | BM284 | 5.65 | | 0.092 | | 0.021 | 1.37 | Cerinza |
|  | 3 | PV131 | 5.09 | | 0.098 | | 0.029 | 0.71 | Cerinza |
|  | 3 | BM197 | 4.20 | | 0.075 | | 0.045 | 0.69 | Cerinza |
|  | 7 | PV242 | 4.28 | | 0.094 | | 0.045 | 0.87 | G10022 |
|  | 7 | BMd40 | 4.98 | | 0.083 | | 0.030 | 0.69 | Cerinza |
|  | 8 | ATA 247 | 4.27 | | 0.096 | | 0.045 | 0.84 | Cerinza |
|  | 9 | BM148 | 5.06 | | 0.090 | | 0.029 | 0.74 | G10022 |
|  | 10 | ATA 76 | 5.45 | | 0.120 | | 0.024 | 1.17 | G10022 |
|  |  |  |  | |  | |  |  |  |
| **K coat** |  |  |  | |  | |  |  |  |
|  | 2 | ATA7 | 7.14 | | 0.111 | | 0.010 | 2940 | Cerinza |
|  | 7 | PV35 | 9.09 | | 0.154 | | 0.004 | 1300 | G10022 |
|  | 8 | PV53 | 7.47 | | 0.122 | | 0.008 | 1350 | G10022 |
|  | 10 | BMd42 | 7.50 | | 0.118 | | 0.008 | 950 | Cerinza |
|  | 1 | PV233 | 6.70 | | 0.116 | | 0.012 | 1200 | G10022 |
|  | 3 | BMc180 | 5.17 | | 0.091 | | 0.027 | 1220 | G10022 |
|  | 4 | PV-ag4 | 6.64 | | 0.110 | | 0.013 | 1300 | Cerinza |
|  | 4 | BMd16 | 5.30 | | 0.084 | | 0.025 | 1820 | Cerinza |
|  | 4 | BM171 | 5.84 | | 0.098 | | 0.019 | 1920 | Cerinza |
|  | 5 | PV93 | 5.41 | | 0.093 | | 0.024 | 1080 | G10022 |
|  | 8 | BMc121 | 5.08 | | 0.087 | | 0.028 | 1260 | G10022 |
|  | 8 | ATA 289 | 4.90 | | 0.086 | | 0.031 | 2190 | Cerinza |
|  | 10 | PV-at5 | 5.46 | | 0.146 | | 0.025 | 1710 | Cerinza |
|  | 11 | BMd41 | 6.09 | | 0.097 | | 0.017 | 1390 | Cerinza |
|  |  |  |  | |  | |  |  |  |
| **K cotyledon** |  |  |  | |  | |  |  |  |
|  | 1 | PV139 | 8.01 | | 0.136 | | 0.007 | 710 | G10022 |
|  | 2 | PV94 | 5.46 | | 0.097 | | 0.023 | 470 | G10022 |
|  | 3 | PV131 | 5.75 | | 0.109 | | 0.020 | 590 | Cerinza |
|  | 3 | BMd36 | 6.56 | | 0.105 | | 0.013 | 610 | G10022 |
|  | 6 | BMd37 | 4.73 | | 0.078 | | 0.034 | 520 | Cerinza |
|  |  |  |  | |  | |  |  |  |
| **Mg coat** |  |  |  | |  | |  |  |  |
|  | 1 | PV233 | 7.29 | | 0.125 | | 0.009 | 380 | Cerinza |
|  | 4 | PV182 | 8.23 | | 0.139 | | 0.006 | 470 | G10022 |
|  | 5 | BMd28 | 7.93 | | 0.126 | | 0.007 | 660 | G10022 |
|  | 6 | BMc220 | 9.74 | | 0.148 | | 0.003 | 400 | Cerinza |
|  | 1 | PVatcc1 | 4.50 | | 0.072 | | 0.038 | 290 | Cerinza |
|  | 3 | AG1 | 6.02 | | 0.100 | | 0.017 | 330 | Cerinza |
|  | 3 | ATA 26 | 4.70 | | 0.095 | | 0.035 | 400 | G10022 |
|  | 4 | BMd9 | 5.32 | | 0.084 | | 0.025 | 780 | G10022 |
|  | 7 | BM210 | 4.56 | | 0.077 | | 0.037 | 280 | G10022 |
|  | 7 | BM183 | 6.20 | | 0.105 | | 0.016 | 590 | Cerinza |
|  | 9 | BM148 | 5.06 | | 0.090 | | 0.029 | 290 | G10022 |
|  | 11 | BMd41 | 5.57 | | 0.089 | | 0.022 | 410 | Cerinza |
|  | 11 | BMc202 | 4.86 | | 0.081 | | 0.032 | 250 | Cerinza |
|  |  |  |  | |  | |  |  |  |
| **Mg cotyledon** | |  |  | |  | |  |  |  |
|  | 5 | BMc321 | 7.33 | | 0.130 | | 0.009 | 140 | G10022 |
|  | 8 | ATA 289 | 11.10 | | 0.176 | | 0.002 | 320 | Cerinza |
|  | 9 | PV149 | 8.79 | | 0.157 | | 0.005 | 130 | Cerinza |
|  | 10 | BMc159 | 10.37 | | 0.166 | | 0.002 | 320 | Cerinza |
|  | 1 | BM221 | 6.45 | | 0.108 | | 0.014 | 150 | Cerinza |
|  | 2 | GAT91 | 5.95 | | 0.098 | | 0.018 | 130 | G10022 |
|  | 3 | BMc333 | 4.43 | | 0.083 | | 0.040 | 120 | G10022 |
|  | 3 | BM159 | 4.79 | | 0.080 | | 0.033 | 80 | Cerinza |
|  | 4 | BMd9 | 5.33 | | 0.084 | | 0.024 | 240 | Cerinza |
|  | 9 | BMd21 | 5.70 | | 0.101 | | 0.021 | 110 | Cerinza |
|  | 10 | BMc274 | 5.66 | | 0.095 | | 0.021 | 90 | Cerinza |
|  |  |  |  | |  | |  |  |  |
| **Mn coat** |  |  |  | |  | |  |  |  |
|  | 11 | BMc368 | 28.08 | | 0.334 | | 0.000 | 2.82 | Cerinza |
|  | 9 | BMc254 | 13.51 | | 0.200 | | 0.001 | 0.67 | Cerinza |
|  | 6 | BMd37 | 9.35 | | 0.143 | | 0.003 | 0.69 | Cerinza |
|  | 9 | BM114 | 12.22 | | 0.203 | | 0.001 | 1.00 | Cerinza |
|  | 9 | BM154 | 7.56 | | 0.117 | | 0.008 | 0.60 | Cerinza |
|  | 10 | PV-at5 | 8.23 | | 0.205 | | 0.007 | 1.09 | Cerinza |
|  | 10 | PV185 | 7.49 | | 0.130 | | 0.008 | 0.89 | Cerinza |
|  | 11 | ATA6 | 12.50 | | 0.248 | | 0.001 | 1.28 | Cerinza |
|  | 3 | PV131 | 6.44 | | 0.121 | | 0.014 | 0.61 | Cerinza |
|  | 3 | BM197 | 5.09 | | 0.089 | | 0.028 | 0.58 | Cerinza |
|  | 6 | BM170 | 6.97 | | 0.112 | | 0.011 | 0.74 | Cerinza |
|  | 8 | PV173 | 5.58 | | 0.104 | | 0.022 | 0.73 | Cerinza |
|  | 10 | BMc5 | 4.52 | | 0.081 | | 0.038 | 0.45 | Cerinza |
|  |  |  |  | |  | |  |  |  |
| **Mn cotyledon** | |  |  | |  | |  |  |  |
|  | 3 | BM181 | 24.71 | | 0.306 | | 0.000 | 3.48 | Cerinza |
|  | 3 | ATA 26 | 24.38 | | 0.351 | | 0.000 | 4.08 | Cerinza |
|  | 9 | ATAME 1 | 19.15 | | 0.281 | | 0.000 | 2.90 | Cerinza |
|  | 3 | BM98 | 12.14 | | 0.181 | | 0.001 | 1.86 | Cerinza |
|  | 4 | BMd9 | 12.25 | | 0.174 | | 0.001 | 5.69 | Cerinza |
|  | 9 | BMd54 | 14.72 | | 0.224 | | 0.000 | 2.42 | Cerinza |
|  | 9 | BM148 | 14.83 | | 0.225 | | 0.000 | 2.32 | Cerinza |
|  | 9 | BM114 | 8.63 | | 0.152 | | 0.005 | 2.10 | Cerinza |
|  | 9 | BMd21 | 10.47 | | 0.170 | | 0.002 | 2.26 | Cerinza |
|  | 1 | BMd76 | 6.69 | | 0.105 | | 0.012 | 2.27 | G10022 |
|  | 2 | PV-cct1 | 4.50 | | 0.123 | | 0.041 | 2.18 | Cerinza |
|  | 3 | BM159 | 6.52 | | 0.106 | | 0.013 | 1.76 | Cerinza |
|  | 4 | PV-at3 | 4.85 | | 0.088 | | 0.032 | 1.75 | Cerinza |
|  | 4 | PV182 | 6.85 | | 0.118 | | 0.012 | 2.30 | Cerinza |
|  | 6 | BMc220 | 4.63 | | 0.076 | | 0.036 | 1.48 | G10022 |
|  | 7 | PV35 | 5.88 | | 0.105 | | 0.019 | 1.71 | Cerinza |
|  | 8 | PV53 | 4.58 | | 0.078 | | 0.037 | 1.71 | Cerinza |
|  | 8 | BMc258 | 5.95 | | 0.103 | | 0.018 | 1.68 | G10022 |
|  |  |  |  | |  | |  |  |  |
| **P coat** |  |  |  | |  | |  |  |  |
|  | 11 | BN | 14.41 | | 0.208 | | 0.000 | 170 | Cerinza |
|  | 3 | PV87 | 8.33 | | 0.129 | | 0.005 | 60 | Cerinza |
|  | 4 | BMd16 | 7.09 | | 0.109 | | 0.010 | 130 | Cerinza |
|  | 4 | PV-at3 | 8.01 | | 0.138 | | 0.007 | 90 | Cerinza |
|  | 4 | PV182 | 9.12 | | 0.152 | | 0.004 | 100 | Cerinza |
|  | 10 | BMd42 | 9.35 | | 0.143 | | 0.003 | 60 | Cerinza |
|  | 1 | PV107 | 6.28 | | 0.104 | | 0.015 | 60 | Cerinza |
|  | 2 | ATA7 | 5.29 | | 0.085 | | 0.025 | 160 | Cerinza |
|  | 3 | ATA 26 | 4.58 | | 0.092 | | 0.037 | 90 | Cerinza |
|  | 4 | PV-ag4 | 5.49 | | 0.092 | | 0.023 | 70 | Cerinza |
|  | 4 | PV-ctt1 | 5.67 | | 0.102 | | 0.021 | 80 | G10022 |
|  | 8 | PV173 | 4.54 | | 0.086 | | 0.038 | 70 | Cerinza |
|  | 9 | PV101 | 4.31 | | 0.084 | | 0.043 | 70 | G10022 |
|  | 10 | PV-at5 | 4.40 | | 0.121 | | 0.043 | 80 | Cerinza |
|  | 10 | PV181 | 4.66 | | 0.089 | | 0.036 | 80 | Cerinza |
|  | 10 | ATA 76 | 6.57 | | 0.141 | | 0.014 | 110 | Cerinza |
|  | 11 | BMd41 | 4.08 | | 0.067 | | 0.048 | 70 | Cerinza |
|  | 11 | BMd22 | 4.26 | | 0.070 | | 0.043 | 100 | Cerinza |
|  |  |  |  | |  | |  |  |  |
| **P cotyledon** |  |  |  | |  | |  |  |  |
|  | 1 | PV133 | 4.66 | | 0.078 | | 0.035 | 390 | G10022 |
|  | 3 | BM181 | 4.79 | | 0.079 | | 0.033 | 380 | Cerinza |
|  | 4 | BM171 | 5.57 | | 0.093 | | 0.022 | 630 | G10022 |
|  | 10 | PV185 | 6.29 | | 0.112 | | 0.015 | 460 | Cerinza |
|  |  |  |  | |  | |  |  |  |
| **S coat** |  |  |  | |  | |  |  |  |
|  | 1 | BMc313 | 8.80 | | 0.158 | | 0.005 | 30 | Cerinza |
|  | 4 | PV-ag4 | 8.19 | | 0.132 | | 0.006 | 40 | Cerinza |
|  | 9 | PV101 | 7.74 | | 0.141 | | 0.008 | 30 | G10022 |
|  | 10 | BMd42 | 7.42 | | 0.117 | | 0.009 | 30 | Cerinza |
|  | 11 | BN | 9.13 | | 0.142 | | 0.004 | 60 | Cerinza |
|  | 1 | BM221 | 4.34 | | 0.076 | | 0.042 | 40 | Cerinza |
|  | 1 | BM284 | 4.15 | | 0.069 | | 0.046 | 40 | Cerinza |
|  | 1 | PV107 | 5.80 | | 0.097 | | 0.019 | 20 | Cerinza |
|  | 1 | PV54 | 4.13 | | 0.075 | | 0.047 | 20 | Cerinza |
|  | 1 | PV233 | 4.25 | | 0.077 | | 0.044 | 30 | Cerinza |
|  | 2 | BM142 | 4.27 | | 0.076 | | 0.044 | 30 | Cerinza |
|  | 3 | PV87 | 5.13 | | 0.084 | | 0.027 | 20 | Cerinza |
|  | 4 | PV-ctt1 | 4.22 | | 0.078 | | 0.045 | 30 | G10022 |
|  | 6 | BMc220 | 4.27 | | 0.071 | | 0.043 | 20 | Cerinza |
|  | 8 | ATA 289 | 6.23 | | 0.107 | | 0.016 | 70 | Cerinza |
|  | 10 | BMc159 | 6.20 | | 0.106 | | 0.016 | 70 | Cerinza |
|  |  |  |  | |  | |  |  |  |
| **S cotyledon** |  |  |  | |  | |  |  |  |
|  | 3 | ATA 26 | 9.69 | | 0.177 | | 0.003 | 280 | Cerinza |
|  | 4 | BMd9 | 8.85 | | 0.132 | | 0.004 | 500 | Cerinza |
|  | 9 | ATAME 1 | 9.25 | | 0.159 | | 0.004 | 210 | Cerinza |
|  | 1 | PV133 | 4.63 | | 0.078 | | 0.036 | 180 | G10022 |
|  | 3 | BM181 | 5.90 | | 0.095 | | 0.018 | 190 | Cerinza |
|  | 3 | BM98 | 5.21 | | 0.087 | | 0.026 | 130 | Cerinza |
|  | 4 | BM171 | 5.74 | | 0.096 | | 0.020 | 290 | G10022 |
|  | 4 | PV182 | 5.32 | | 0.094 | | 0.025 | 200 | Cerinza |
|  | 4 | ATA 143 | 6.90 | | 0.117 | | 0.011 | 190 | Cerinza |
|  | 9 | ATA9 | | 5.16 | | 0.092 | 0.027 | 380 | Cerinza |
