# Supplementary material for: A legume biofortification quandary: variability and genetic control of seed coat micronutrient accumulation in common beans
Source: Front Plant Sci. 2013 Jul 29;4:275. doi: 10.3389/fpls.2013.00275 (PMC3725406; doi:10.3389/fpls.2013.00275)
Supplement: Supplementary file 2 [file 50458_Blair_DataSheet2.DOCX]

**Supplementary Table 2.** Genotypes selected from the advanced backcross (AB) population for quantitative trait loci (QTL) analysis and their average seed coat and cotyledonary mineral concentrations compared to their whole seed concentration.

| **Genotype** | **Seed coat concentration (ppm^1^)**  **averages (n=2)** | | | | | | | | | | **Cotyledonary concentration (ppm^1^) –**  **averages (n=2)** | | | | | | | | | | **Whole seed concentration (ppm^1^) - averages (n=4)** | |
| --- | --- | --- | --- | --- | --- | --- | --- | --- | --- | --- | --- | --- | --- | --- | --- | --- | --- | --- | --- | --- | --- | --- |
| **Mineral^2^** | **B** | **Ca** | **Cu** | **Fe** | **K** | **Mg** | **Mn** | **P** | **S** | **Zn** | **B** | **Ca** | **Cu** | **Fe** | **K** | **Mg** | **Mn** | **P** | **S** | **Zn** | **Fe** | **Zn** |
| **AB-QTL-2** | 12.1 | 14585 | 1.3 | **206.9** | 9665 | 3015 | 3 | 575 | 360 | **22.9** | 13.2 | 245 | 6.5 | **60.3** | 13905 | 1445 | 13.9 | 4275 | 2030 | **28.3** | 60.1 | 27.4 |
| **AB-QTL-3** | 10.7 | 17385 | 1.8 | **251.8** | 7200 | 3375 | 2.9 | 540 | 350 | **33.6** | 12.2 | 250 | 8.1 | **59.6** | 13880 | 1610 | 14.3 | 4520 | 2245 | **31.3** | 80.5 | 34.6 |
| **AB-QTL-5** | 15.9 | 18765 | 3.2 | **134.1** | 8990 | 2990 | 3.1 | 530 | 425 | **18.4** | 15.2 | 265 | 7.9 | **55** | 15000 | 1805 | 17 | 4620 | 2190 | **30.6** | 64.5 | 28.5 |
| **AB-QTL-6** | 17 | 17620 | 1.2 | **37.21** | 7350 | 2815 | 2.9 | 380 | 325 | **38.8** | 14.7 | 280 | 6.8 | **75.8** | 13575 | 1520 | 13.8 | 4495 | 2170 | **32.5** | 73.7 | 30.1 |
| **AB-QTL-7** | 19.9 | 14740 | 1.8 | **43.8** | 7655 | 3090 | 3.8 | 435 | 385 | **36.9** | 39 | 320 | 6.4 | **77.2** | 13880 | 1560 | 15.7 | 4130 | 1960 | **27.6** | 65.0 | 29.8 |
| **AB-QTL-10** | 11.4 | 18605 | 1.8 | **238.9** | 8025 | 2600 | 3.4 | 525 | 360 | **30.4** | 14.1 | 310 | 9.5 | **73.3** | 14505 | 1715 | 21.6 | 5540 | 2780 | **41.7** | 81.0 | 31.9 |
| **AB-QTL-11** | 14.6 | 15240 | 1.3 | **42.4** | 5950 | 3290 | 3.2 | 390 | 325 | **29.4** | 13.1 | 240 | 7.8 | **79.2** | 13645 | 1610 | 15.3 | 4555 | 2260 | **32.4** | 77.0 | 32.1 |
| **AB-QTL-13** | 10.7 | 16760 | 0.9 | **45.94** | 5520 | 3475 | 3.7 | 325 | 310 | **44.3** | 12 | 260 | 7.3 | **82.4** | 14125 | 1605 | 17.5 | 4955 | 2365 | **33.4** | 74.2 | 31.5 |
| **AB-QTL-14** | 17.6 | 15485 | 3.6 | **54.52** | 7120 | 3380 | 4.5 | 435 | 355 | **49** | 14 | 275 | 8.7 | **86.5** | 13600 | 1390 | 15.8 | 5095 | 2495 | **35.9** | 70.2 | 27.9 |
| **AB-QTL-16** | 16.6 | 19010 | 1.5 | **42.47** | 6935 | 3160 | 3.1 | 465 | 400 | **32.6** | 32.6 | 275 | 8.4 | **81.4** | 13740 | 1615 | 15.9 | 4690 | 2145 | **31.9** | 66.5 | 26.7 |
| **AB-QTL-19** | 12.8 | 15865 | 1.3 | **26.08** | 6525 | 3615 | 2.8 | 370 | 320 | **27.3** | 12.9 | 260 | 7.7 | **70.2** | 13015 | 1565 | 14.6 | 4310 | 2295 | **29.9** | 67.1 | 28.5 |
| **AB-QTL-28** | 8.36 | 16605 | 1.6 | **31.82** | 7410 | 3445 | 4.2 | 395 | 305 | **17.5** | 14.7 | 335 | 9.1 | **70.9** | 13655 | 1695 | 15.5 | 4660 | 2260 | **37.1** | 66.5 | 28.7 |
| **AB-QTL-29** | 7.71 | 19715 | 1.5 | **32.67** | 6730 | 2790 | 3.1 | 375 | 310 | **33.4** | 14.5 | 340 | 8.3 | **77.4** | 14135 | 1500 | 14.5 | 4405 | 2460 | **33.2** | 82.9 | 29.7 |
| **AB-QTL-30** | 14.1 | 16970 | 1.6 | **31.89** | 6490 | 3615 | 2.6 | 395 | 335 | **29.9** | 14 | 255 | 7.5 | **69** | 13625 | 1555 | 14.9 | 4660 | 2130 | **29.9** | 69.5 | 28.8 |
| **AB-QTL-33** | 14.9 | 17500 | 1.8 | **26.53** | 8085 | 2795 | 2.4 | 365 | 335 | **23.9** | 22 | 375 | 8 | **79.7** | 14430 | 1525 | 14.9 | 4700 | 2090 | **32** | 73.7 | 28.9 |
| **AB-QTL-42** | 7.33 | 20325 | 1.4 | **253.1** | 2700 | 3395 | 3.4 | 450 | 365 | **55.3** | 25.3 | 325 | 7.4 | **77.5** | 15235 | 1405 | 21.1 | 5425 | 2720 | **39.6** | 95.8 | 37.9 |
| **AB-QTL-49** | 15 | 20355 | 1.9 | **35.75** | 5035 | 3720 | 2.7 | 365 | 340 | **36.1** | 21.7 | 330 | 8.7 | **84.6** | 14060 | 1425 | 16.6 | 4905 | 2330 | **33.4** | 74.2 | 29.1 |
| **AB-QTL-50** | 16.8 | 16790 | 1.2 | **38.11** | 6190 | 3345 | 2.6 | 380 | 365 | **25.8** | 24.5 | 295 | 7.7 | **88** | 14335 | 1435 | 16.9 | 4365 | 2195 | **31.2** | 86.0 | 31.4 |
| **AB-QTL-55** | 11.9 | 16470 | 1.4 | **34** | 5885 | 3675 | 3 | 360 | 330 | **36** | 18 | 270 | 8.4 | **81.6** | 15015 | 1525 | 15.1 | 4895 | 2150 | **32.5** | 84.0 | 30.8 |
| **AB-QTL-57** | 14.1 | 17430 | 1.2 | **33.99** | 5230 | 3195 | 2.8 | 385 | 320 | **33.8** | 15.2 | 280 | 7.8 | **80.3** | 13530 | 1335 | 16.4 | 4545 | 2180 | **30.8** | 83.8 | 33.4 |
| **AB-QTL-61** | 12.8 | 20620 | 1.5 | **28.87** | 8275 | 2770 | 3 | 415 | 360 | **30.7** | 17.4 | 330 | 7.8 | **77.4** | 15405 | 1525 | 17.9 | 4625 | 2140 | **30.4** | 71.2 | 31.1 |
| **AB-QTL-66** | 16.4 | 17195 | 1.1 | **38.43** | 7520 | 3390 | 3 | 415 | 340 | **21.9** | 15.3 | 260 | 7.3 | **80** | 14530 | 1360 | 14.1 | 4590 | 2210 | **33.1** | 72.6 | 29.8 |
| **AB-QTL-67** | 21.3 | 16935 | 2 | **31.55** | 7185 | 3435 | 3.4 | 425 | 355 | **30.8** | 21.3 | 270 | 8.8 | **77.5** | 14740 | 1455 | 15.1 | 4620 | 2250 | **30.4** | 74.3 | 30.0 |
| **AB-QTL-70** | 10 | 18185 | 1.1 | **241.5** | 4625 | 3845 | 2.8 | 485 | 350 | **49.5** | 14.9 | 220 | 8.6 | **61.8** | 15155 | 1410 | 16.1 | 5390 | 2285 | **35.1** | 82.8 | 32.5 |
| **AB-QTL-72** | 12.7 | 15805 | 1.1 | **24.47** | 6430 | 3375 | 3.5 | 330 | 315 | **37.2** | 18.1 | 285 | 7.9 | **76.2** | 15320 | 1520 | 17.4 | 4930 | 2275 | **31.8** | 65.1 | 30.4 |
| **AB-QTL-73** | 14.4 | 15875 | 1 | **29.18** | 6715 | 3155 | 2.3 | 380 | 330 | **20.9** | 17.2 | 255 | 7.4 | **76.1** | 14265 | 1485 | 15 | 4045 | 2115 | **28.1** | 69.4 | 29.1 |
| **AB-QTL-78** | 11.4 | 19585 | 1.9 | **190.7** | 7415 | 2980 | 3.6 | 515 | 395 | **30.5** | 16 | 295 | 9.4 | **67.5** | 14230 | 1415 | 17.5 | 5065 | 2470 | **37.7** | 80.3 | 33.9 |
| **AB-QTL-79** | 14.4 | 14625 | 1.4 | **20.42** | 7165 | 4450 | 3.7 | 455 | 390 | **26.6** | 17.3 | 190 | 6.9 | **65.2** | 13995 | 1520 | 16 | 4395 | 2170 | **28.6** | 64.5 | 27.7 |
| **AB-QTL-80** | 14.3 | 14460 | 1.3 | **29.98** | 7255 | 3535 | 3.2 | 380 | 340 | **33.6** | 14.9 | 235 | 8 | **76.8** | 14855 | 1520 | 14.3 | 4765 | 2155 | **31.2** | 82.9 | 30.2 |
| **AB-QTL-81** | 13.5 | 15740 | 1.7 | **34.41** | 6320 | 3500 | 3.1 | 350 | 320 | **46.6** | 14.1 | 230 | 8.6 | **81.9** | 15380 | 1520 | 14.7 | 4740 | 2255 | **33.9** | 84.7 | 33.4 |
| **AB-QTL-87** | 13.9 | 16475 | 2 | **48.76** | 6815 | 3655 | 4.1 | 390 | 370 | **36.5** | 16.8 | 235 | 9.3 | **78.3** | 15790 | 1655 | 17.3 | 5000 | 2415 | **33.2** | 83.4 | 33.5 |
| **AB-QTL-88** | 17 | 13145 | 1.7 | **33.2** | 7765 | 3380 | 6 | 450 | 360 | **49.4** | 15.5 | 190 | 9.2 | **77** | 15270 | 1380 | 15.7 | 5505 | 2335 | **33.2** | 73.3 | 32.5 |
| **AB-QTL-90** | 14.8 | 17670 | 1.2 | **28.43** | 6655 | 3280 | 2.9 | 370 | 330 | **26.4** | 16.8 | 255 | 7.2 | **71.9** | 14965 | 1510 | 14.3 | 4730 | 2170 | **29.9** | 74.3 | 29.4 |
| **AB-QTL-92** | 15.3 | 20175 | 1.7 | **35.82** | 5895 | 2970 | 3 | 355 | 355 | **43.8** | 13.9 | 235 | 9.5 | **72.7** | 14800 | 1395 | 15.6 | 4825 | 2355 | **32.1** | 67.4 | 32.8 |
| **AB-QTL-93** | 14.6 | 16785 | 1.4 | **42.49** | 7775 | 3250 | 3.2 | 385 | 315 | **33.8** | 16.6 | 305 | 7.6 | **82.1** | 14780 | 1490 | 15.2 | 4455 | 2085 | **31.3** | 80.4 | 30.9 |
| **AB-QTL-94** | 17.3 | 15850 | 1.6 | **32.06** | 7515 | 3140 | 3.1 | 405 | 375 | **45.9** | 14.5 | 255 | 8.2 | **73.6** | 14745 | 1265 | 14.1 | 4715 | 2020 | **31.5** | 73.4 | 34.5 |
| **AB-QTL-95** | 18.8 | 17825 | 1.6 | **32.21** | 6310 | 3675 | 2.9 | 375 | 325 | **34.3** | 16 | 265 | 9 | **76.9** | 15075 | 1605 | 16.4 | 4880 | 2240 | **31.4** | 77.8 | 30.7 |
| **AB-QTL-99** | 17.1 | 17945 | 1 | **28.45** | 6240 | 3195 | 2.8 | 405 | 320 | **21** | 16.9 | 315 | 6.8 | **72.9** | 14145 | 1390 | 14.3 | 4130 | 1915 | **28** | 81.6 | 32.2 |
| **AB-QTL-100** | 16.7 | 16420 | 1.6 | **36.83** | 7150 | 2980 | 3.7 | 360 | 350 | **45.9** | 16.7 | 285 | 8.9 | **81** | 15560 | 1500 | 18.5 | 5160 | 2445 | **36.9** | 80.7 | 32.9 |
| **AB-QTL-101** | 11.9 | 16450 | 2 | **263.3** | 8680 | 3325 | 4.3 | 600 | 380 | **32.6** | 17.2 | 250 | 8.7 | **57.8** | 15415 | 1495 | 17.2 | 4995 | 2340 | **33.2** | 82.9 | 31.1 |
| **AB-QTL-103** | 16.4 | 17320 | 1.6 | **31.06** | 7060 | 3330 | 2.4 | 405 | 350 | **35.2** | 22.2 | 300 | 8.3 | **70.6** | 15560 | 1570 | 17.8 | 4670 | 2240 | **31.2** | 68.2 | 30.9 |
| **AB-QTL-104** | 18.7 | 15740 | 1.7 | **37.82** | 7035 | 3680 | 3.9 | 405 | 355 | **37.6** | 15.2 | 285 | 7.9 | **79** | 14855 | 1430 | 16.1 | 5200 | 2300 | **32.9** | 73.7 | 33.3 |
| **AB-QTL-106** | 14.4 | 16945 | 1.6 | **32.87** | 6490 | 3435 | 4.3 | 360 | 310 | **45.7** | 13.6 | 295 | 9.4 | **81** | 15085 | 1480 | 17.9 | 5680 | 2525 | **36.3** | 80.4 | 33.0 |
| **AB-QTL-107** | 15.7 | 17350 | 1.5 | **26.29** | 6195 | 3290 | 2.7 | 370 | 310 | **25.8** | 14 | 335 | 8 | **75** | 14420 | 1395 | 16.2 | 4695 | 2205 | **31.4** | 74.1 | 28.2 |
| **AB-QTL-108** | 12.4 | 17875 | 1.1 | **31.93** | 6285 | 3295 | 3.3 | 365 | 325 | **25.8** | 13.1 | 315 | 6.5 | **73.4** | 14580 | 1530 | 18.1 | 4525 | 2060 | **29.2** | 65.3 | 28.1 |
| **AB-QTL-115** | 17.5 | 16485 | 1.2 | **28.55** | 6555 | 3135 | 2.2 | 390 | 305 | **29.4** | 16.9 | 345 | 7.7 | **88.1** | 14855 | 1475 | 13.8 | 5050 | 2405 | **34.2** | 80.4 | 28.8 |
| **AB-QTL-116** | 7.63 | 15725 | 1.9 | **237.9** | 5040 | 3890 | 3.2 | 480 | 370 | **32.4** | 13.1 | 245 | 7.9 | **67.3** | 15015 | 1610 | 16 | 5195 | 2415 | **34.1** | 77.1 | 31.9 |
| **AB-QTL-122** | 10.6 | 14450 | 2.4 | **59.89** | 6150 | 3690 | 2.5 | 470 | 385 | **30.8** | 13.4 | 330 | 8.3 | **85.2** | 13705 | 1300 | 12.5 | 4780 | 2270 | **33.1** | 81.9 | 28.4 |
| **AB-QTL-123** | 12.2 | 17175 | 1.7 | **51.54** | 6230 | 2645 | 3.1 | 400 | 345 | **29.6** | 14.3 | 345 | 7.4 | **79.2** | 14065 | 1445 | 17 | 4795 | 2380 | **33.3** | 74.3 | 32.3 |
| **AB-QTL-127** | 10.9 | 16345 | 2.1 | **43.59** | 6015 | 3445 | 2.9 | 400 | 335 | **30.8** | 13.8 | 310 | 8.6 | **84.5** | 14130 | 1470 | 15.3 | 5235 | 2285 | **34.2** | 83.8 | 28.8 |
| **AB-QTL-129** | 11 | 17640 | 2 | **31.12** | 6485 | 3840 | 4.1 | 370 | 335 | **33.1** | 12.1 | 270 | 9.7 | **74** | 14010 | 1460 | 18.2 | 5815 | 2595 | **31.9** | 67.5 | 28.0 |
| **AB-QTL-130** | 11.3 | 15650 | 2 | **32.99** | 8150 | 4080 | 3.4 | 540 | 430 | **38.1** | 13.4 | 230 | 9.3 | **79.9** | 14265 | 1320 | 15.8 | 5465 | 2540 | **35.5** | 68.1 | 29.0 |
| **AB-QTL-131** | 11.3 | 17355 | 1.9 | **29.31** | 8475 | 3795 | 3.2 | 495 | 390 | **35.4** | 13.7 | 245 | 8.6 | **77.7** | 14340 | 1375 | 15.3 | 5015 | 2450 | **34.9** | 62.9 | 28.3 |
| **AB-QTL-132** | 14.3 | 18590 | 2.6 | **31.71** | 7335 | 3650 | 2.8 | 485 | 425 | **30.4** | 13.5 | 300 | 8 | **74.1** | 14065 | 1415 | 13.3 | 4595 | 2120 | **29.8** | 66.1 | 31.2 |
| **AB-QTL-133** | 11.2 | 18495 | 1.4 | **218.4** | 7455 | 3350 | 3.5 | 635 | 390 | **28** | 13.2 | 290 | 6.9 | **64.2** | 14115 | 1425 | 19.2 | 4760 | 2440 | **35.5** | 74.5 | 30.0 |
| **AB-QTL-135** | 13.7 | 20030 | 1.6 | **35.97** | 5920 | 3580 | 2.9 | 425 | 335 | **30.3** | 12.2 | 330 | 8.4 | **93.6** | 13310 | 1325 | 15.9 | 5155 | 2465 | **35.8** | 80.3 | 34.3 |
| **AB-QTL-138** | 14.3 | 16745 | 1.5 | **30.8** | 6935 | 3330 | 2.8 | 420 | 335 | **27.7** | 13.1 | 360 | 7.1 | **74.7** | 13810 | 1360 | 13.5 | 4475 | 2205 | **31.3** | 70.7 | 31.3 |
| **AB-QTL-139** | 12.4 | 16760 | 2.3 | **30.07** | 4645 | 3440 | 2.7 | 340 | 340 | **22.2** | 14.6 | 315 | 7.9 | **88** | 14930 | 1545 | 15.2 | 4705 | 2260 | **31.8** | 79.8 | 30.3 |
| **AB-QTL-142** | 16.7 | 19820 | 1.9 | **48.32** | 7060 | 3530 | 3.1 | 475 | 365 | **23.5** | 12.2 | 335 | 8.7 | **81.3** | 13945 | 1500 | 15.7 | 5120 | 2645 | **36.5** | 81.8 | 34.1 |
| **AB-QTL-143** | 8.6 | 15450 | 1.8 | **258.9** | 6840 | 3460 | 3.5 | 530 | 340 | **42.9** | 12.7 | 255 | 7.9 | **70.4** | 15080 | 1490 | 17.4 | 5570 | 2350 | **37.8** | 73.8 | 34.0 |

.

^1^Mineral concentrations given in part per million (ppm); ^2^ Mineral abbreviations: B=boron, Ca=calcium, Cu=copper, Fe=iron, K=potassium, Mg=magnesium, Mn=manganese, P=phosphorus, S=sulfur, Zn=zinc.
